# Ultrasound measurements of superficial and deep masticatory muscles in various postures: reliability and influencers

**DOI:** 10.1038/s41598-020-71378-z

**Published:** 2020-09-01

**Authors:** Pei-Hsuan Chang, Yunn-Jy Chen, Ke-Vin Chang, Wei-Ting Wu, Levent Özçakar

**Affiliations:** 1 Department of Dentistry, School of Dentistry, National Taiwan University Hospital, Taipei, Taiwan, ROC; 2 Department of Physical Medicine and Rehabilitation and Community and Geriatric Research Center, National Taiwan University Hospital, Bei-Hu Branch and National Taiwan University College of Medicine, Taipei, Taiwan, ROC; 3 Department of Physical and Rehabilitation Medicine, Hacettepe University Medical School, Ankara, Turkey

**Keywords:** Anatomy, Health care, Medical research

## Abstract

Masticatory muscle thickness provides objective measurements of the oral motor function, which may change in patients with oral myofascial pain. In this study, we aimed to establish a reliable ultrasound (US) protocol for imaging the superficial and deep masticatory muscles and to identify the potential influencers of the measurements. Forty-eight healthy participants without orofacial pain were enrolled. The intra-and inter-rater reliabilities of US measurements for masseter, temporalis, and lateral pterygoid muscles were assessed. Intraclass correlation coefficients for all muscles were greater than 0.6. The generalised estimating equation was used to analyse the impact of age, gender, laterality, and body mass index on the measurements, whereby age and body mass index were likely to be associated with an increase in masticatory muscle thickness. The thickness tended to be lesser in females. Laterality seemed to exert minimal influence on masticatory muscle thickness. Our study shows acceptable reliability of US in the evaluation of superficial and deep masticatory muscle thickness. Future studies are warranted to validate the usefulness of US imaging in patients with oral myofascial pain syndrome.

## Introduction

Temporomandibular disorder (TMD) is frequently observed in patients seeking dental treatment. It comprises various findings concerning the stomatognathic system, involving the temporomandibular joints, masticatory muscles, teeth, and ears^1,2^. The prevalence of TMDs in the general population ranges from 34.9 to 42.4% across different studies^3,4^, and the incidence is more prevalent in females^5^. Of note, oral myofascial pain is commonplace in about 10–15% of patients with TMD^6^.

The aetiology of oral myofascial pain is still controversial. The proposed mechanisms include overuse of the masticatory muscles and a decrease in its pain threshold following central sensitization^7^. While overuse might lead to hypertrophy of the masticatory muscles in the early stages, persistent pain could result in disuse atrophy in chronic cases^8^. In this sense, masticatory muscle thickness provides objective measurements of the oral motor function^9^, which is supposed to change in patients suffering from oral myofascial pain.

Several imaging modalities, such as computed tomography (CT), magnetic resonance imaging (MRI), and ultrasound (US) have been used to image the masticatory muscles. CT allows precise delineation of the skull bone where the masticatory muscles attach. However, radiation and poor resolution of the soft tissues make it unsuitable for muscle thickness measurements. Nowadays, MRI serves as the gold standard for depicting soft tissue pathologies^10^, but it is limited by its high cost and contraindications in patients with metal implants. US has various advantages over CT and MRI, i.e. real-time evaluation, zero radiation, cost-effectiveness, portability, and ease of dynamic examination^11^. Further, it is capable of delineating muscle fibres—making it highly effective for imaging muscle trauma and neuromuscular diseases^12–15^.

Although several studies^16,17^ have assessed masticatory muscle thickness using US, only a few of them considered the influence of potential confounders, such as laterality, oral posture, age, gender, weight and height^18,19^. Additionally, previous research focused only on the superficial masticatory muscles but not the deep ones, which would be equally important for the oral motor function. Therefore, the present study aimed to develop a reliable US protocol for imaging the superficial and deep masticatory muscles and to identify the potential influencers of muscle thickness measurements.

## Materials and methods

### Participants

This pilot study included healthy adults (24 males and 24 females) aged more than 20 years who did not experience any oral myofascial pain, TMD, and sleep bruxism. All the subjects underwent a physical examination in accordance with the Diagnostic Criteria for Temporomandibular Disorders^1^ and were not allowed to have pain in the jaw, temple, and auricular region. Subjects without complete dentition were excluded. All participants were required to submit an informed consent and the study protocol was approved by the institutional review board of National Taiwan University Hospital, Taipei, Taiwan. (201804014RINA). All methods were carried out in accordance with relevant guidelines and regulations (the Declaration of Helsinki).

### Ultrasound measurements

The BenQ Ultrasound System (T3300, BenQ Medical Technology Corp., Taipei, Taiwan) was used for the measurements. The superficial masticatory muscles were assessed using a linear probe (L154BH, 4–15 MHz), while a curved probe (C62B, 2–6 MHz) was employed to obtain the images of the lateral pterygoid muscle due to its deep location. The scanning depth was set at 40 mm for measuring the masseter and the temporalis muscles, and at 60 mm for surveying the lateral pterygoid muscle. The frame rate was set at 300 frames per second.

The subjects were seated on a chair with back support while their heads were kept in a neutral position. The hands were comfortably rested on the knees. All the examinations were repeated three times for each muscle at each position, and the average values were used for the analyses. The measurements were conducted offline by using the stored US pictures and imaging processing software, Image J (National Institutes of Health, Rockville Pike, Bethesda, MD).

**Figure 1:**
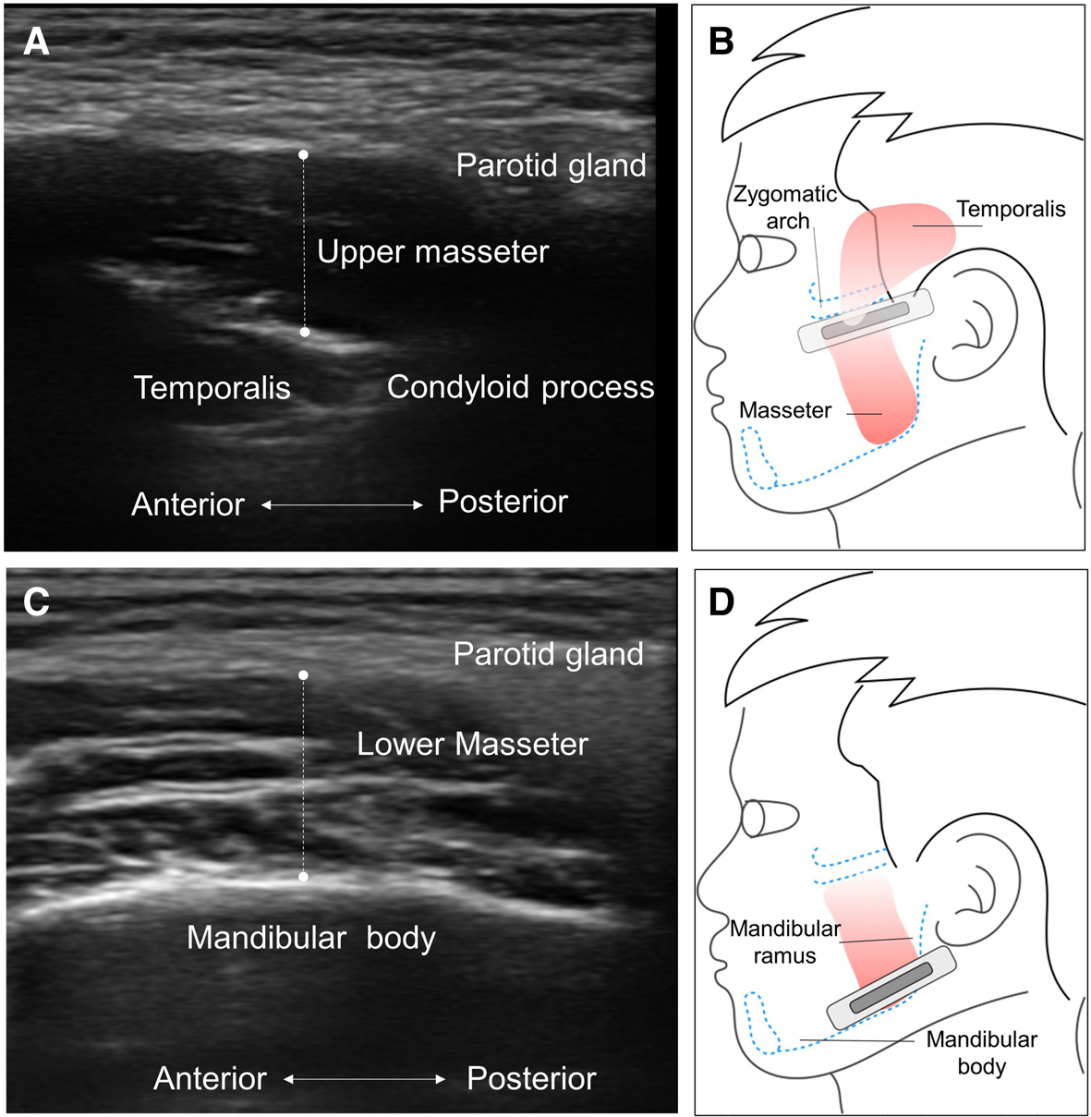
Ultrasound imaging (**A**) and transducer placement (**B**) for scanning the upper masseter muscle. Ultrasound imaging (**C**) and transducer placement (**D**) for scanning the lower masseter muscle. Note that the length of the dashed lines indicates the thickness of the muscle.

**Figure 2:**
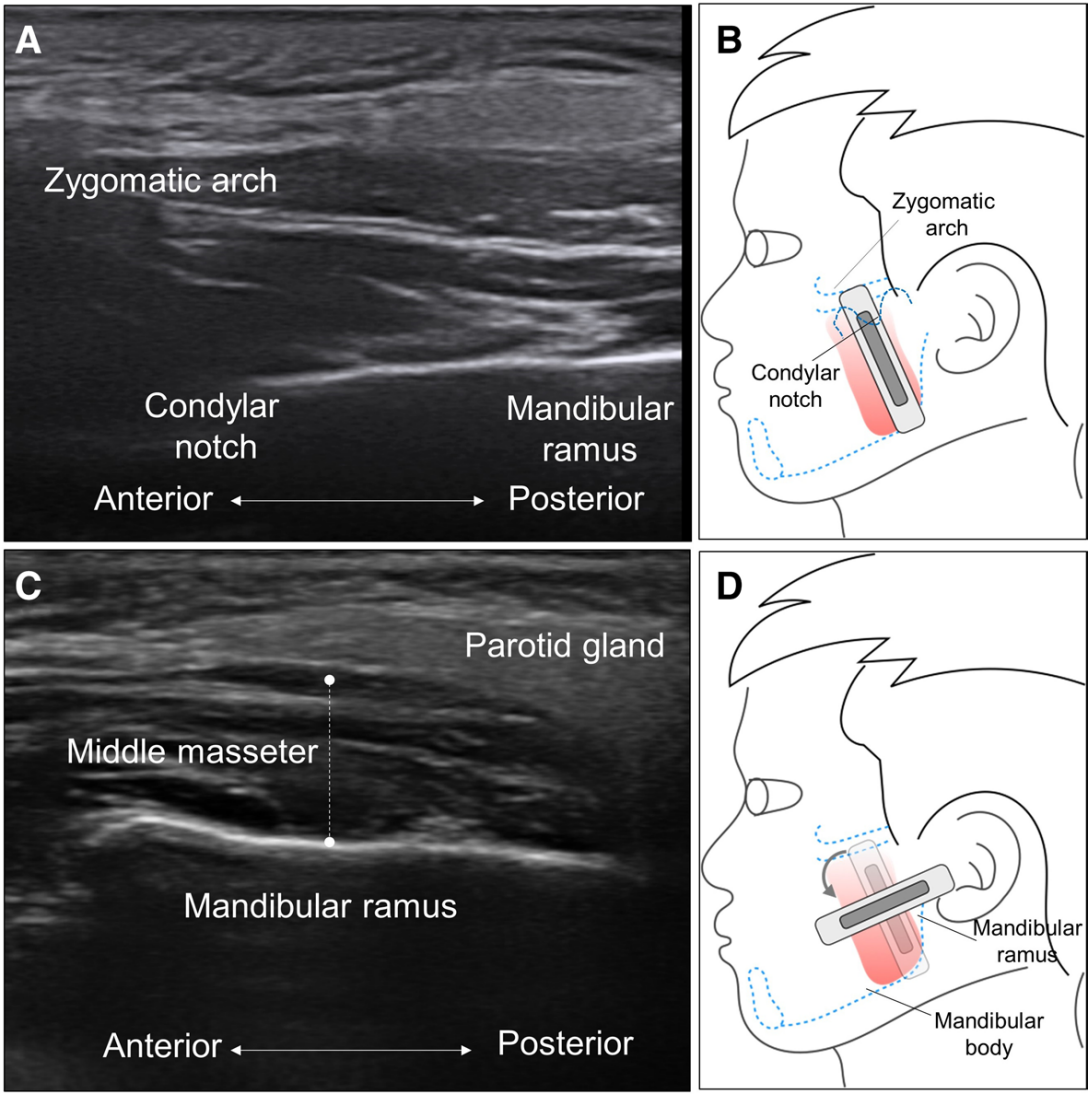
Ultrasound imaging (**A**) when the transducer is placed in the coronal plane bridging the condylar notch (**B**). Ultrasound imaging (**C**) of the middle masseter muscle when the transducer is rotated to the horizontal plane (**D**). Note that the length of the dashed lines indicates the thickness of the muscle.

The thickness of the masseter muscle was measured at its upper, middle, and lower parts. To visualize the upper part, the transducer was first placed along the zygomatic arch and then slightly moved towards the chip until the zygomatic arch became invisible. It was then relocated towards the chin parallel to the long axis of the mandibular body, near the angular portion of the mandibular ramus to inspect the lower masseter (Fig. 1). The transducer was then rotated 90 degrees to locate the tip of the condylar notch, following which it was pivoted back to the plane parallel to the long axis of the mandibular body using the above-mentioned point as the centre of rotation to measure its middle part (Fig. 2). The muscle thickness was defined as the maximal distance between the outer and inner fasciae. The muscle was measured bilaterally during relaxation, maximal jaw clenching, and maximal mouth opening. During clenching, we asked our participants to bite as forceful as possible without feeling pain over the teeth. During maximal mouth opening, we required our participants to open the mouths as wide as possible without have discomfort over the temporomandibular joints.

**Figure 3:**
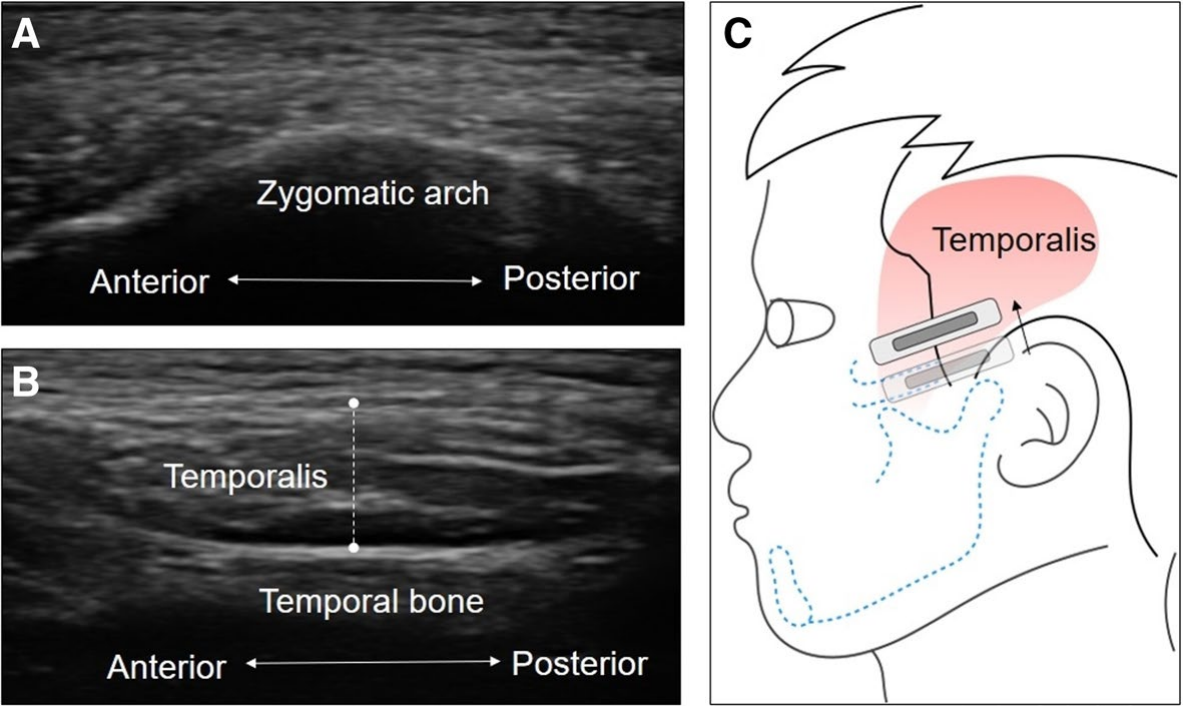
Ultrasound imaging of the zygomatic arch (**A**) and the temporalis muscle (**B**) as the transducer is moved towards the temporal fossa (**C**). Note that the length of the dashed lines indicates the thickness of the muscle.

For the temporalis muscle, the linear probe was placed on the upper border of the zygomatic arch and slightly moved cranially and parallel to the short axis of the zygomatic arch until the temporalis muscle was shown on the screen (Fig. 3). The muscle thickness was defined as the maximal distance between the outer and inner fasciae of the temporalis muscle. The muscle was also measured bilaterally during relaxation, maximal jaw clenching, and maximal mouth opening.

**Figure 4:**
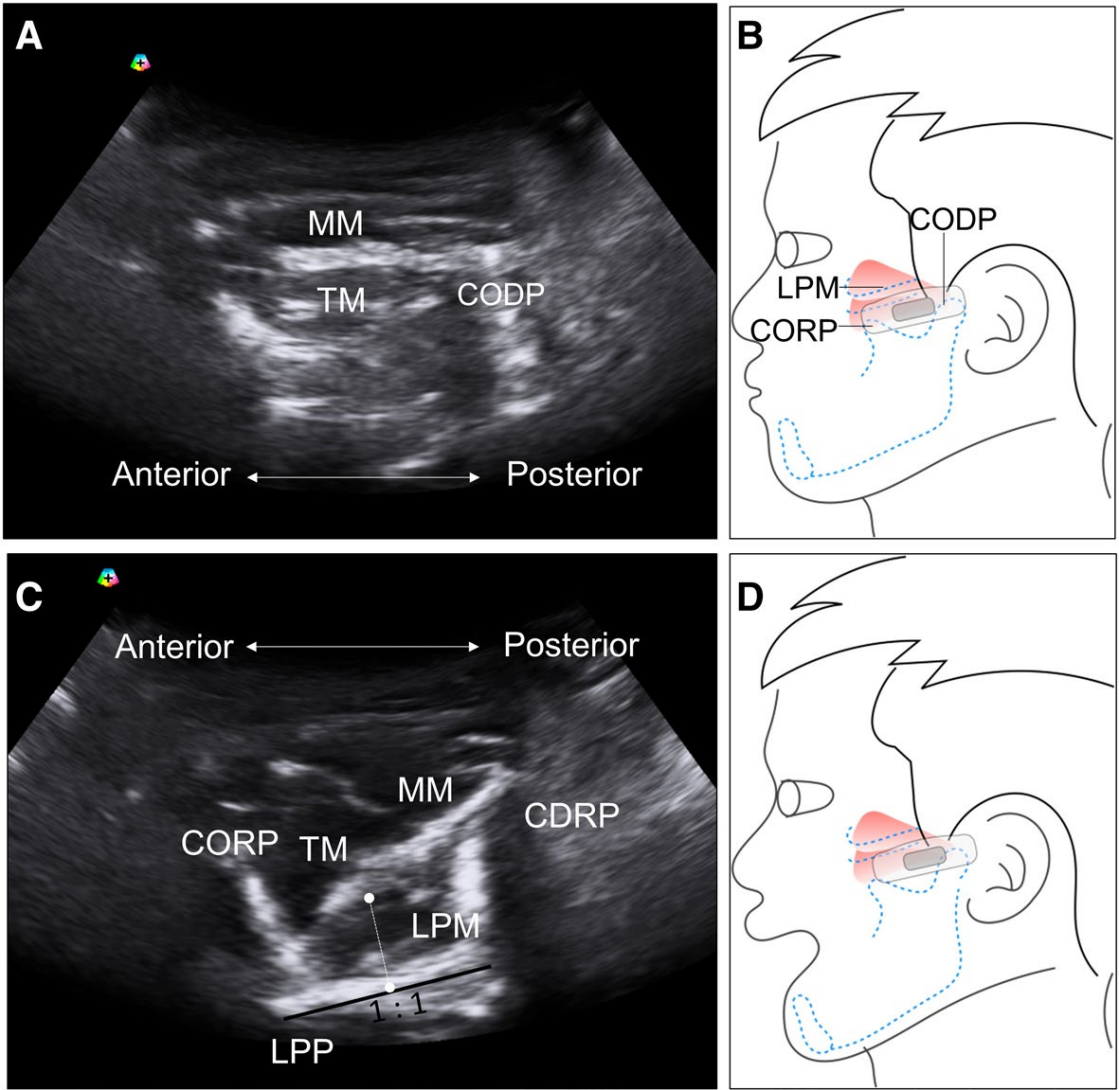
Ultrasound imaging (**A**) when the transducer is placed in the horizontal plane caudal to the zygomatic arch with the mouth closed (**B**). Ultrasound imaging (**C**) of the lateral pterygoid muscle (LPM) when the transducer is placed in the horizontal plane caudal to the zygomatic arch with the mouth opened (**D**). The length of the dashed lines indicates the thickness of the muscle. MM, masseter muscle; CORP, coronoid process; CODP, condylar process; TM, temporalis muscle; LPM, lateral pterygoid muscle.

For the lateral pterygoid muscle, the transducer was placed along the zygomatic arch then relocated caudally to the mandibular notch in the horizontal plane, where a hypoechoic gap can be identified between the coronoid and condylar processes of the mandible^20^. After opening the mouth, the lateral pterygoid muscle was fully observed as a triangular-shaped muscle attached to the lateral pterygoid plate (Fig. 4). The distance between the outer and inner fasciae in the middle of the lateral pterygoid muscle was defined as its thickness. The scanning protocol of the three muscles was summarized in supplementary Table 1.

**Table 1:** Intra- and inter-rater evaluations and corresponding differences of ultrasound muscle thickness measurements.

	1st rater 1st evaluation in mm (95% CI)	1st rater 2nd evaluation in mm (95% CI)	2nd rater 1st evaluation in mm (95% CI)	Intra-rater mean difference in mm (95% CI)	*p* value	Inter-rater mean difference in mm (95% CI)	*p* value
Upper masseter (relaxation)	15.48 ± 1.75 (14.71 to 16.25)	15.45 ± 1.81 (14.66 to 16.24)	14.78 ± 1.59 (14.08 to 15.48)	− 0.02 ± 0.94 (− 0.43 to 0.39)	0.907	− 0.69 ± 0.35 (− 1.04 to − 0.34)	0.001*
Upper masseter (clenching)	16.26 ± 1.88 (15.43 to 17.09)	16.20 ± 1.83 (15.40 to 17.00)	15.53 ± 1.62 (14.82 to 16.24)	− 0.01 ± 0.96 (− 0.43 to 0.41)	0.784	− 0.73 ± 1.11 (− 1.21 to − 0.25)	0.008*
Upper masseter (maximal opening)	16.10 ± 2.15 (15.16 to 17.04)	15.91 ± 1.78 (15.13 to 16.69)	15.99 ± 1.87 (15.17 to 16.81)	− 0.27 ± 1.55 (− 0.95 to 0.41)	0.562	− 0.11 ± 0.58 (− 0.69 to 0.47)	0.719
Middle masseter (relaxation)	14.85 ± 1.66 (14.12 to 15.58)	14.85 ± 2.37 (13.81 to 15.89)	13.67 ± 1.99 (12.80 to 14.54)	0.00 ± 1.23 (− 0.54 to 0.54)	0.981	− 1.18 ± 1.40 (− 1.79 to − 0.57)	0.001*
Middle masseter (clenching)	15.63 ± 1.87 (14.81 to 16.45)	15.65 ± 2.40 (14.6 0to 16.70)	14.85 ± 2.06 (13.95 to 15.75)	− 0.09 ± 1.16 (− 0.6 to 0.42)	0.937	− 0.78 ± 1.35 (− 1.37 to − 0.19)	0.018*
Middle masseter (maximal opening)	14.69 ± 2.17 (13.74 to 15.64)	14.93 ± 2.25 (13.94 to 15.92)	13.99 ± 2.13 (13.06 to 14.92)	0.28 ± 1.25 (− 0.27 to 0.83)	0.403	− 0.70 ± 1.33 (− 1.28 to − 0.12)	0.029*
Lower masseter (relaxation)	11.12 ± 2.52 (10.01 to 12.23)	12.03 ± 2.63 (10.88 to 13.18)	11.72 ± 2.69 (10.54 to 12.90)	0.85 ± 1.85 (0.04 to 1.66)	0.040*	0.60 ± 2.03 (− 0.29 to 1.49)	0.202
Lower masseter (clenching)	11.53 ± 2.57 (10.4 to 12.66)	12.57 ± 2.75 (11.36 to 13.78)	12.52 ± 2.57 (11.4 to 13.64)	0.96 ± 2.10 (0.04 to 1.88)	0.039*	1.00 ± 1.96 (0.14 to 1.86)	0.035*
Lower masseter (maximal opening)	11.56 ± 2.12 (10.63 to 12.49)	11.95 ± 2.75 (10.75 to 13.15)	11.57 ± 2.54 (10.46 to 12.68)	0.37 ± 1.38 (− 0.23 to 0.97)	0.223	0.01 ± 1.31 (− 0.56 to 0.58)	0.981
Temporalis (relaxation)	5.09 ± 0.79 (4.74 to 5.44)	5.14 ± 0.69 (4.84 to 5.44)	5.16 ± 1.07 (4.69 to 5.63)	0.05 ± 0.57 (− 0.2 to 0.3)	0.466	0.08 ± 0.63 (− 0.31 to 0.47)	0.599
Temporalis (clenching)	5.12 ± 0.76 (4.79 to 5.45)	5.09 ± 0.62 (4.82 to 5.36)	5.25 ± 1.03 (4.80 to 5.70)	− 0.03 ± 0.49 (− 0.25 to 0.19)	0.759	0.12 ± 0.50 (− 0.10 to 0.34)	0.364
Temporalis (maximal opening)	4.92 ± 0.69 (4.62 to 5.22 )	5.08 ± 0.64 (4.8 to 5.36)	5.38 ± 1.16 (4.87 to 5.89)	0.16 ± 0.43 (− 0.03 to 0.35)	0.107	0.46 ± 0.78 (0.12 to 0.80)	0.006*
Lateral pterygoid (maximal opening)	14.07 ± 0.84 (13.7 to 14.44)	14.00 ± 1.08 (13.53 to 14.47)	14.37 ± 1.10 (13.89 to 14.85)	− 0.43 ± 0.53 (− 0.66 to − 0.20)	0.705	0.30 ± 0.30 (0.00 to 0.60)	0.070

*CI* confidence interval. The mean differences were calculated using the values from the 1st evaluation of the 1st rater as the references.

*p < 0.05.

### Study flow

Intra-and inter-rater reliability were assessed in the first 10 participants. The 1st session of muscle measurement was conducted by the primary investigator. The 2nd session was conducted one hour later by another investigator using the same scanning protocol, and the 3rd session was conducted by the primary investigator one week after the 1st session. The data derived from the 1st and 2nd sessions were used for analyses of the inter-rater reliability, whereas those of the 1st and 3rd were used for the intra-rater reliability. Examination of the remaining participants was carried out by the primary investigator. Both investigators were board-certificated dentists with more than one-year training in musculoskeletal US. All the participants were recruited based on their designated age (20–40, 40–60, > 60) and sex (female: male ratio = 1:1) stratification.

### Sample size calculation

The sample size was calculated based on a previous study investigating the thickness of masseter muscles in patients with and without temporomandibular joint dysfunction^21^. We assumed that a variation among certain demographic factors led to a mean difference of 0.9 mm in muscle thickness with a standard deviation of 0.5 mm. The number of participants with that variation was hypothesized to be the same as those without. The alpha level was set at 0.05 with a power of 80%. Considering a drop-out rate of 20%, the total number needed was 38.

### Statistical analysis

The data of intra-and inter-rater reliability were analysed by the two-way random effect model and were expressed by the intra-class correlation coefficient (ICC) and its 95% confidence interval (CI). The standard error of measurement (SEM) was calculated using the following formula: SEM = the standard deviation pooled from both evaluations $$\times \sqrt{(1-\mathrm{ICC})}$$^22^. The minimal detectable change (MDC) was obtained employing the following equation: MDC = 1.96 $$\times \sqrt{2}$$$$\times$$ SEM^22^. The paired t-test compared the mean differences between the evaluations of different sessions. The above-mentioned analyses were conducted by using MedCalc (version 14, Ostend, Belgium).

The generalised estimating equation (GEE) was used to analyse the impact of age, gender, laterality, and body mass index (BMI) on the measurements of masticatory muscle thickness. Compared with either height or weight, BMI provides a simple numeric measure of a person’s thickness or thinness and is associated with skeletal muscle mass^23^. Therefore, in studies related to evaluation of muscle quantity^23,24^, BMI is a common parameter used for adjustment. Furthermore, if we put height, weight and BMI in the same model for adjustment, the association of muscle thickness with the body size might not be correctly estimated due to collinearity of weight or height with BMI. The GEE—suitable for managing the clustered or correlated data—was performed using the SPSS (Version 12.0. Chicago, SPSS Inc) software. The participant identification was treated as the clustering variable, whereas laterality (right/left) served as an exchangeable correlation structure. A *p* value less than 0.05 was considered to be statistically significant.

### Ethical approval

All participants were required to submit an informed consent and the study protocol was approved by the institutional review board of National Taiwan University Hospital, Taipei, Taiwan. (201804014RINA). All methods were carried out in accordance with relevant guidelines and regulations (the Declaration of Helsinki).

## Results

The average age and body mass index values of the participants were 46.19 ± 15.94 (standard deviation, SD) years and 23.07 ± 3.14 (SD) kg/m^2^, respectively. Repeated evaluations of the same raters revealed significant differences between the lower masseter thickness measurements during relaxation (0.85 ± 1.85 mm, *p* = 0.040) and jaw clenching (0.96 ± 2.10 mm, *p* = 0.039). Similarly, repeated evaluations of different raters revealed significant differences between the thickness measurements of the upper masseters during relaxation (− 0.69 ± 0.35 mm, *p* = 0.001) and jaw clenching (− 0.73 ± 1.11 mm, *p* = 0.008); middle masseters during relaxation (− 1.18 ± 1.40 mm, *p* = 0.001), jaw clenching (− 0.78 ± 1.35 mm, *p* = 0.018), and maximal mouth opening (− 0.70 ± 1.33 mm, *p* = 0.029); lower masseters during jaw clenching (1.00 ± 1.96 mm, *p* = 0.035); and temporalis muscles during maximal mouth opening (0.46 ± 0.78 mm, *p* = 0.006) (Table 1).

All intra-and inter-rater reliabilities were greater than 0.6, which is considered good according to the Cicchetti’s classification^25^. The intra-rater SEM and MDC ranged from 0.31 mm (temporalis muscles during maximal mouth opening) to 1.49 mm (lower masseters during jaw clenching) and from 0.85 mm (temporalis muscles during maximal mouth opening) to 4.13 mm (lower masseters during jaw clenching), respectively. The inter-rater SEM and MDC ranged from 0.42 mm (temporalis muscles during maximal mouth opening) to 1.44 mm (lower masseters in relaxation) and from 1.16 mm (temporalis muscle during maximal mouth opening) to 3.98 mm (lower masseters during relaxation), respectively (Table 2).

**Table 2:** Intra- and inter-rater reliability of ultrasound muscle thickness measurements.

	Intra-rater ICC (95% CI)	Intra-rater SEM (mm)	Intra-rater MDC (mm)	Inter-rater ICC (95% CI)	Inter-rater SEM (mm)	Inter-rater MDC (mm)
Upper masseter (relaxation)	0.86 (0.68 to 0.94)	0.66	1.84	0.89 (0.74 to 0.95)	0.56	1.55
Upper masseter (clenching)	0.88 (0.71 to 0.95)	0.65	1.81	0.80 (0.56 to 0.92)	0.78	2.17
Upper masseter (maximal opening)	0.73 (0.43 to 0.88)	1.03	2.85	0.78 (0.52 to 0.91)	0.94	2.62
Middle masseter (relaxation)	0.82 (0.61 to 0.93)	0.86	2.39	0.71 (0.40 to 0.87)	0.99	2.74
Middle masseter (clenching)	0.83 (0.62 to 0.93)	0.89	2.47	0.77 (0.50 to 0.90)	0.95	2.64
Middle masseter (maximal opening)	0.85 (0.65 to 0.94)	0.87	2.41	0.85 (0.655 to 0.94)	0.94	2.61
Lower masseter (relaxation)	0.75 (0.47 to 0.89)	1.29	3.59	0.81 (0.58 to 0.92)	1.44	3.98
Lower masseter (clenching)	0.69 (0.36 to 0.86)	1.49	4.13	0.71 (0.40 to 0.87)	1.39	3.85
Lower masseter (maximal opening)	0.84 (0.64 to 0.93)	0.99	2.73	0.84 (0.65 to 0.94)	0.92	2.56
Temporalis (relaxation)	0.70 (0.38 to 0.87)	0.41	1.13	0.78 (0.52 to 0.91)	0.44	1.23
Temporalis (clenching)	0.75 (0.47 to 0.89)	0.35	0.97	0.79 (0.54 to 0.91)	0.42	1.16
Temporalis (maximal opening)	0.79 (0.54 to 0.91)	0.31	0.85	0.76 (0.49 to 0.90)	0.50	1.38
Lateral pterygoid (maximal opening)	0.63 (0.28 to 0.84)	0.58	1.62	0.75 (0.47 to 0.89)	0.49	1.36

*ICC* intra-class correlation coefficient, *SEM* standard error of measurement, *MDC* minimal detectable change.

According to the GEE analysis, age was likely to be positively associated with the masticatory muscle thickness. Statistically significant differences were detected in the lower masseters in all the examined positions (*p* = 0.017, relaxation; *p* = 0.003, jaw clenching; and *p* = 0.010, maximal mouth opening) and only in the maximal mouth opening position (*p* = 0.006) for the lateral pterygoid muscles. Female sex was found to be inversely associated with masticatory muscle thickness; a statistically significant inverse association was observed for the upper masseters during jaw clenching (*p* = 0.034) and middle masseters during maximal mouth opening (*p* = 0.039). The influence of laterality was only seen in the measurement of upper masseters during clenching (*p* = 0.018) whereby the muscle appeared significantly thicker on the left side. BMI was found to be positively associated with the thickness all masticatory muscles whereby significant differences were detected in the temporalis muscle thickness measurements in all examined positions (*p* = 0.008, relaxation; *p* = 0.011, jaw clenching; and *p* = 0.013, maximal mouth opening) (Table 3).

**Table 3:** Association of demographics with masticatory muscle thicknesses (expressed by the beta coefficients and their 95% confidence intervals).

	Upper masseter (relaxation)	Upper masseter (clenching)	Upper masseter (maximal opening)	Middle masseter (relaxation)	Middle masseter (clenching)	Middle masseter (maximal opening)	Lower masseter (relaxation)	Lower masseter (clenching)	Lower masseter (maximal opening)	Temporalis (relaxation)	Temporalis (clenching)	Temporalis (maximal opening)	Lateral pterygoid (maximal opening)
Age (year)	− 0.02 (− 0.05 to 0.01)	− 0.02 (− 0.05 to 0.01)	0.01 (− 0.025 to 0.047)	< 0.001 (− 0.03 to 0.03)	< 0.001 (− 0.03 to 0.03)	0.02 (− 0.01 to 0.06)	0.05 (0.01 to 0.08)	0.06 (0.02 to 0.10)	0.05 (0.01 to 0.09)	0.01 (0.00 to 0.02)	0.01 (0.00 to 0.02)	0.01 (0.00 to 0.02)	0.02 (0.01 to 0.04)
	*p* = 0.120	*p* = 0.206	*p* = 0.536	*p* = 0.972	*p* = 0.907	*p* = 0.230	*p* = 0.017*	*p* = 0.003*	*p* = 0.010*	*p* = 0.106	*p* = 0.282	*p* = 0.079	*p* = 0.006*
Female gender	− 0.81 (− 1.86 to 0.24)	− 1.23 (− 2.37 to − 0.09)	− 1.08 (− 2.477 to 0.311)	− 1.12 (− 2.30 to 0.06)	− 1.10 (− 2.40 to 0.20)	− 1.52 (− 2.97 to − 0.08)	− 1.02 (− 2.44 to 0.40)	− 1.30 (− 2.90 to 0.29)	− 1.34 (− 2.97 to 0.29)	− 0.29 (− 0.63 to 0.06)	− 0.34 (− 0.68 to 0.01)	− 0.31 (− 0.66 to 0.04)	− 0.74 (− 1.32 to − 0.16)
	*p* = 0.132	*p* = 0.034*	*p* = 0.128	*p* = 0.063	*p* = 0.098	*p* = 0.039*	*p* = 0.158	*p* = 0.108	*p* = 0.108	*p* = 0.101	*p* = 0.056	*p* = 0.079	*p* = 0.013*
Left side	0.59 (0.10 to 1.08)	0.39 (− 0.03 to 0.80)	0.23 (− 0.185 to 0.648)	0.02 (− 0.41 to 0.44)	− 0.20 (− 0.69 to 0.29)	− 0.05 (− 0.50 to 0.40)	0.26 (− 0.22 to 0.73)	0.01 (− 0.57 to 0.59)	− 1.34 (− 2.97 to 0.29)	− 0.11 (− 0.25 to 0.04)	− 0.04 (− 0.20 to 0.11)	− 0.12 (− 0.26 to 0.01)	0.03 (− 0.22 to 0.29)
	*p* = 0.018*	*p* = 0.071	*p* = 0.277	*p* = 0.936	*p* = 0.420	*p* = 0.829	*p* = 0.285	*p* = 0.970	*p* = 0.108	*p* = 0.145	*p* = 0.583	*p* = 0.065	*p* = 0.805
Body mass index	0.13 (− 0.03 to 0.28)	0.15 (− 0.02 to 0.31)	0.11 (− 0.071 to 0.295)	0.16 (− 0.02 to 0.34)	0.15 (− 0.05 to 0.35)	0.14 (− 0.07 to 0.34)	0.16 (− 0.09 to 0.42)	0.14 (− 0.14 to 0.41)	0.10 (− 0.17 to 0.37)	0.07 (0.02 to 0.12)	0.07 (0.02 to 0.01)	0.07 (0.01 to 0.12)	0.01 (− 0.09 to 0.11)
	*p* = 0.106	*p* = 0.078	*p* = 0.232	*p* = 0.081	*p* = 0.153	*p* = 0.191	*p* = 0.212	*p* = 0.339	*p* = 0.459	*p* = 0.008*	*p* = 0.011*	*p* = 0.013*	*p* = 0.842

*p < 0.05.

## Discussion

The present study employed a standard US scanning protocol to measure masticatory muscle thickness yielding good intra-and inter-rater reliability. To the authors’ best knowledge, this is the first study exploring the reliability of US measurements for the lateral pterygoid muscle. During the literature search, only a few articles had investigated the influence of age, gender, height and weight on superficial masticatory muscles^18,19^. In the present research, the multivariate analysis also revealed that age and BMI were likely to be associated with an increase in masticatory muscle thickness, whereas the thickness tended to be lesser in females. Laterality was observed to exert minimal influence on masticatory muscle thickness.

Our research demonstrates that the reliability of US measurements of masticatory muscle thickness, represented by intraclass correlation coefficients (ICCs), ranges from 0.69 to 0.89. Lin et al.^26^ examined the reliability of MRI measurements for the masseter muscle and found better inter-and intra-rater ICCs (0.996 and 0.997, respectively) when compared with those of ours. However, US provides real-time and dynamic assessment of masticatory muscles as well as a more accessible and cost-effective option than MRI. In 2003, Emshoff et al*.*^27^ used US to measure the facial and neck muscles and found ICCs ranging from 0.70 to 0.92. In 2019, Barotsis et al.^28^ conducted another US study for assessing the masseter thickness and the range of ICCs was between 0.295 and 0.991. While the ICCs from our data are not inferior to those from previous US literature, our results also include muscle thickness measurements in various masticatory postures.

In the present study, we utilized several methods to ensure the reliability of US measurements for the masticatory muscles in different mouth positions. First, bony landmarks, such as the zygomatic arch, mandibular ramus, mandibular body and mandibular notch were used to standardize the site of measurement, which also facilitated the examiner to repeat the scanning process on different participants. Second, the masseter muscles were measured at various segments to minimize the influence of the intramuscular tendon near the insertion on the mandible. In our study, we identified a lower ICC value regarding thickness measurements of the lower masseters during clenching (Table 2). The potential cause might be derived from their muscle origin (the superficial and deep heads). As we did not control the anterior-to-posterior dimension during examination, the variation of contribution from both heads of the masseters might affect the reliability of measurements. Herein, this issue cautions investigators regarding the increased variability observed while measuring the distal parts of the superficial masticatory muscles.

The lateral pterygoid muscle plays a crucial role in controlling protrusion, depression, and unilateral movement of the lower jaw^20^. While its upper part originates from the greater wing of the sphenoid bone, its lower part originates from the lateral surface of the lateral pterygoid plate. Both parts insert onto the neck of the mandible. Measurement of its thickness using US has never been performed until now. There are three reasons why its thickness measurement is difficult. First, it is deeply located and is not easily appreciated by a linear transducer. Second, it is obscured by the mandibular ramus when the mouth is closed. Third, it is triangular-shaped—challenging the thickness definition. Therefore, we implemented three approaches to make the measurement plausible. First, we used the curved transducer to improve the penetration of US beam for better visualization of the deep structures. Second, the open-mouth view was utilized to avoid the acoustic shadowing of the mandibular ramus. Third, the thickness of the lateral pterygoid muscle was clearly defined by using the midpoint of its superficial fascia. To this end, our study showed that the reliability of thickness measurements for the lateral pterygoid muscle could be as good as those for the superficial masticatory muscles.

As shown in Table 2, the MDC values of the middle and the lower masseter muscles were larger than the other masticatory muscles. A substantial portion of the middle and lower masseter muscles has been evolved to form the tendinous component, causing an increase in variability of muscle thickness across different measurements as well as in the corresponding MDC values. The masseter muscle consists two major heads, superficial and deep^29^. The superficial head is located more anteriorly and arises from a tendinous aponeurosis. In contrast, the deep head originates from the posterior portion of the zygomatic arch and appears more muscular than the superficial head. The portions of muscle fibers from each head can affect the measurement of the maximal muscle thickness. In this study, the muscle was measured at its thickest portion on the US images without considering possible variations in the anterior-to-posterior dimension, which also accounted for larger MDC values of the middle and lower masseter muscles.

The GEE analysis revealed a likely positive association between the masticatory muscle thickness and age, i.e. statistically significant for the lower masseters and lateral pterygoid muscles. Likewise, a previous US study reported that masticatory muscle thickness gradually increased with age in a population younger than 60 years^19^. This finding might be attributed to age-related stature changes and hypertrophy due to repeated use.

In the present study, we did not specifically measure the echogenicity of the masticatory muscle. As the present study included measurements of the deep muscles, the gain of ultrasound signals needed to be adjusted dynamically to improve the visibility of the deep muscle fascia. Therefore, the measurements of muscle echogenicity might not be reliable using our study design. However, muscle echogenicity usually increases with aging due to fat replacement^30^. Isolated thickness measurement was shown to be less informative than muscle echogenicity in patients with neuromuscular disease^31^. Therefore, future studies can be designed to specifically evaluate the echogenicity of the masticatory muscles, which would be beneficial for exploration of age- or disease-related alternations of muscle texture.

Regarding sex-related differences, males were observed to have thicker masticatory muscles than females, especially for the masseters and lateral pterygoid muscles. Our analysis also revealed a positive association independent of age, body stature, and laterality. One possible explanation would be the relationship between the masticatory muscle thickness and craniofacial morphology, especially for the masseter muscle^32,33^. Males tend to have greater facial length than females^34^. Moreover, the diameters of type II muscle fibres are larger than type I^35^, and there is a higher portion of type II muscle fibres in male masseters as compared to those of female^34^.

Our study also identified a likely positive association between BMI and masticatory thickness, especially for the temporalis muscle. Since the temporalis muscle travels a longer distance on the skull than the masseter and lateral pterygoid muscles, its size is more dependent on the head volume. Similarly, an antecedent anthropometric study identified a high correlation between brain volume and BMI—partially supporting this issue^36^.

Our findings uncovered minimal influence of laterality on the masticatory muscle thickness. As this result is consistent with a previous study^37^ investigating similar issues, it would be interesting to examine whether asymmetry in muscle thickness can be found in patients with masticatory problems in future studies.

There were several limitations that need to be acknowledged. First, the present study used a cross-sectional design. Whether the changes observed in masticatory muscle thickness were associated with long-term health outcomes could not be determined through our analysis. Second, the zygomatic bone is the main obstacle in observing the lateral pterygoid muscle using US. MRI or CT would be needed if the investigators intend to measure its thickness in the closed-mouth position. Third, as the present research aimed to establish the reference standards of masticatory muscle thickness, only asymptomatic volunteers were enrolled. Future studies are needed to explore whether the masticatory muscle thicknesses are altered in patients with TMD or oral myofascial pain syndrome.

In conclusion, using a standardized protocol, US can be employed to evaluate the thickness of superficial and deep masticatory muscles with acceptable reliability. These values may vary in populations with different age range, gender, and BMI values. Future studies are warranted to validate the usefulness of US imaging in patients with common clinical syndromes, such as TMD, malocclusion of teeth, oral motor dysphagia and oral myofascial pain syndrome.

## Supplementary information

Supplementary information
