# Supplementary material for: Ultrasound measurements of superficial and deep masticatory muscles in various postures: reliability and influencers
Source: Sci Rep. 2020 Sep 1;10:14357. doi: 10.1038/s41598-020-71378-z (PMC7463001; doi:10.1038/s41598-020-71378-z)
Supplement: Supplementary file 1 — Supplementary information [file 41598_2020_71378_MOESM1_ESM.docx]

**Ultrasound Measurements of Superficial and Deep Masticatory Muscles in Various Postures: Reliability and Influencers**

Pei-Hsuan Chang ^1^, Yunn-Jy Chen ^1^, Ke-Vin Chang ^2^, Wei-Ting Wu ^2^, Levent Özçakar ^3^

^1^ Department of Dentistry, School of Dentistry, National Taiwan University Hospital Taipei, Taiwan, ROC

^2^ Department of Physical Medicine and Rehabilitation and Community and Geriatric Research Center, National Taiwan University Hospital, Bei-Hu Branch and National Taiwan University College of Medicine, Taipei, Taiwan, ROC

^3^ Department of Physical and Rehabilitation Medicine, Hacettepe University Medical School, Ankara, Turkey

**Corresponding author:** Ke-Vin Chang, MD, PhD

Department of Physical Medicine and Rehabilitation, National Taiwan University Hospital, Bei-Hu Branch and National Taiwan University College of Medicine, Taipei, Taiwan, ROC

**Email:** kvchang011@gmail.com

**Supplementary Table 1.** Ultrasound measurement protocol for the superficial and deep masticatory muscles

| **Muscle** | **Participant’s posture** | **Transducer placement** | **Muscle Thickness Measurement** |
| --- | --- | --- | --- |
| Masseter muscle | Sitting on a chair with back support while their heads were kept in a neutral position | **Upper part**: the transducer was first placed along the zygomatic arch and then slightly moved towards the chip until the zygomatic arch became invisible.  **Middle part**: the transducer is then relocated towards the chin parallel to the long axis of the mandibular body.  **Lower part**: the transducer is then rotated 90 degrees to locate the tip of the condylar notch, following which it is pivoted back to the plane parallel to the long axis of the mandibular body | The muscle thickness is defined as the maximal distance between the outer and inner fasciae. The muscle is measured bilaterally during relaxation, maximal jaw clenching, and maximal mouth opening. |
| Temporalis muscle | As the above-described posture | The transducer is placed on the upper border of the zygomatic arch and slightly moved cranially and parallel to the short axis of the zygomatic arch until the temporalis muscle was shown on the screen | The muscle thickness is defined as the maximal distance between the outer and inner fasciae of the temporalis muscle. The muscle was also measured bilaterally during relaxation, maximal jaw clenching, and maximal mouth opening. |
| Lateral pterygoid muscle | As the above-described posture | The transducer is placed along the zygomatic arch then relocated caudally to the mandibular notch in the horizontal plane. After opening the mouth, the lateral pterygoid muscle was fully observed as a triangular-shaped muscle attached to the lateral pterygoid plate | The distance between the outer and inner fasciae in the middle of the lateral pterygoid muscle is defined as its thickness. The muscle is also measured bilaterally maximal mouth opening. |
